# Orthopædic Surgery

**Published:** 1900-09

**Authors:** 


**Orthopædic Surgery.** By J. Jackson Clarke, M.B. Pp. xix., 454. London: Cassell and Company, Limited. 1899.

The opportunities for studying deformities of children are so abundant in London that it is not to be wondered at that surgeons attached to the various orthopædic hospitals should from time to time produce fresh text-books on orthopædic surgery. Perhaps an undue number of such books have lately appeared, but the one before us is certainly one of the best. It may be that there is not much that is new in the volume. It seems to be reserved for a few men like William Adams, Macewen, Hoffa, Lorenz, or Lewis Sayre to open up new work in orthopædics, while the majority of us must be content to work along the lines they have indicated, and perfect in various minutiæ the proceedings advised by such pioneers.

Mr. Jackson Clarke seems to have made himself well acquainted with modern developments in orthopædics, and displays an eminently fair and judicial spirit in dealing with the work of others. He is particularly cautious on the subject of forcible reduction in tubercular deformities of the spine, contenting himself by saying that while further experience is required before any final decision can be made, yet that a mechanical method of fixing the spine in the over-extended position is better than the plaster-of-Paris occipito-pelvic jacket, while cases treated from the beginning by efficient mechanical means will give as good results as those treated by forcible extension.

In the treatment of congenital talipes equino-varus we note with approval that he is not such an advocate for free tenotomy as many others, but pays much attention to constant and frequent mouldings and manipulations to correct the deformity. He rightly rejects the severe cutting operations, such as Buchanan's or Phelps's, for all except the severe relapsing cases of older children, but in our opinion the author much undervalues the use of plaster-of-Paris splints in young children.

The author gives us a good account of the symptoms and treatment of that peculiarly American affection "metatarsalgia," or "Morton's painful foot," but we are rather surprised to find no mention of excision of a branch of the external plantar nerve which has proved so efficacious in the hands of many surgeons.

The book is well illustrated, and is written in a clear and lucid manner. It will form an admirable students' text-book.

